# Supplementary material for: Resveratrol Promotes Tumor Microvessel Growth via Endoglin and Extracellular Signal-Regulated Kinase Signaling Pathway and Enhances the Anticancer Efficacy of Gemcitabine against Lung Cancer
Source: Cancers (Basel). 2020 Apr 15;12(4):974. doi: 10.3390/cancers12040974 (PMC7225973; doi:10.3390/cancers12040974)
Supplement: Supplementary file 1 [file cancers-12-00974-s001.pdf]

Supplementary Material

Resveratrol Promotes Tumor Microvessel Growth via Endoglin and Extracellular Signal-Regulated Kinase Signaling Pathway and Enhances the Anticancer Efficacy of Gemcitabine against Lung Cancer

San-Hai Qin, Andy T. Y. Lau, Zhan-Ling Liang, Heng Wee Tan, Yan-Chen Ji, Qiu-Hua Zhong, Xiao-Yun Zhao and Yan-Ming Xu

Table S1. Human Angiogenesis Array coordinates.

Coordinate	Target/Control	Gene ID	Alternate Nomenclature	Coordinate	Target/Control	Gene ID	Alternate Nomenclature
A1, A2	Reference Spots	N/A	—	D1, D2	MIP-1α	6348	CCL3
A5, A6	Activin A	3624	—	D3, D4	MMP-8	4317	—
A7, A8	ADAMTS-1	9510	—	D5, D6	MMP-9	4318	—
A9, A10	Angiogenin	283	ANG	D7, D8	NRG1-β1	3084	HRG1-β1
A11, A12	Angiopoietin-1	284	Ang-1	D9, D10	Pentraxin 3 (PTX3)	5806	TSG-14
A13, A14	Angiopoietin-2	285	Ang-2	D11, D12	PD-ECGF	1890	—
A15, A16	Angiostatin/Plasminogen	5340	—	D13, D14	PDGF-AA	5154	—
A17, A18	Amphiregulin	374	AR	D15, D16	PDGF-AB/PDGF-BB	5155	—
A19, A20	Artemin	9048	—	D17, D18	Persephin	5623	—
A23, A24	Reference Spots	N/A	—	D19, D20	Platelet Factor 4 (PF4)	5196	CXCL4
B1, B2	Coagulation Factor III	2152	TF	D21, D22	PlGF	5228	—
B3, B4	CXCL16	58191	—	D23, D24	Prolactin	5617	—
B5, B6	DPPIV	1803	CD26	E1, E2	Serpin B5	5268	Maspin
B7, B8	EGF	1950	—	E3, E4	Serpin E1	5054	PAI-1
B9, B10	EG-VEGF	84432	PK1	E5, E6	Serpin F1	5176	PEDF
B11,B12	Endoglin	2022	CD105	E7, E8	TIMP-1	7076	—
B13, B14	Endostatin/Collagen XVIII	80781	—	E9, E10	TIMP-4	7079	—
B15, B16	Endothelin-1	1906	ET-1	E11, E12	Thrombospondin-1	7057	TSP-1
B17, B18	FGF acidic	2246	FGF-1	E13, E14	Thrombospondin-2	7058	TSP-2
B19, B20	FGF basic	2263	FGF-2	E15, E16	uPA	5328	—
B21, B22	FGF-4	2249	—	E17, E18	Vasohibin	22846	—
B23, B24	FGF-7	2252	KGF	E19, E20	VEGF	7422	—
C1, C2	GDNF	2668	—	E21, E22	VEGF-C	7424	—
C3, C4	GM-CSF	1437	—	F1, F2	Reference Spots	N/A	—
C5, C6	HB-EGF	1839	—	F23, F24	Negative Control	N/A	Control (−)
C7, C8	HGF	3082	—				
C9, C10	IGFBP-1	3484	—				
C11, C12	IGFBP-2	3485	—				
C13, C14	IGFBP-3	3486	—				
C15, C16	IL-1β	3553	IL-1F2				
C17, C18	IL-8	3576	CXCL8				
C19, C20	LAP (TGF-β1)	7040	—				
C21, C22	Leptin	3952	—				
C23, C24	MCP-1	6347	CCL2				

Human Angiogenesis Array Coordinates

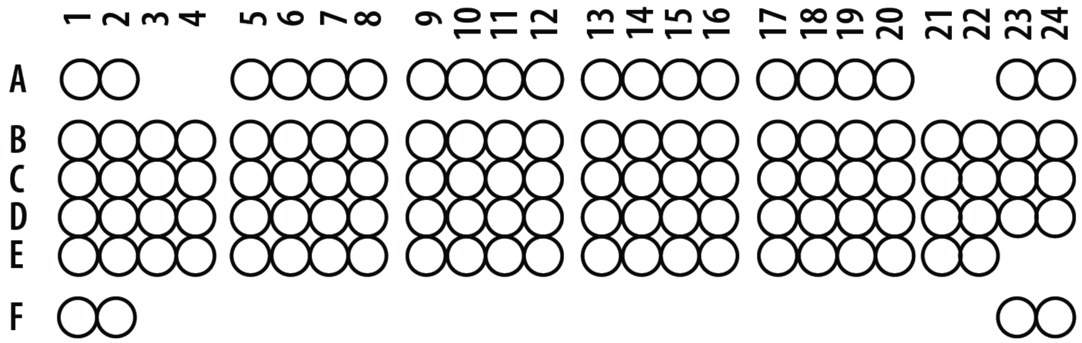

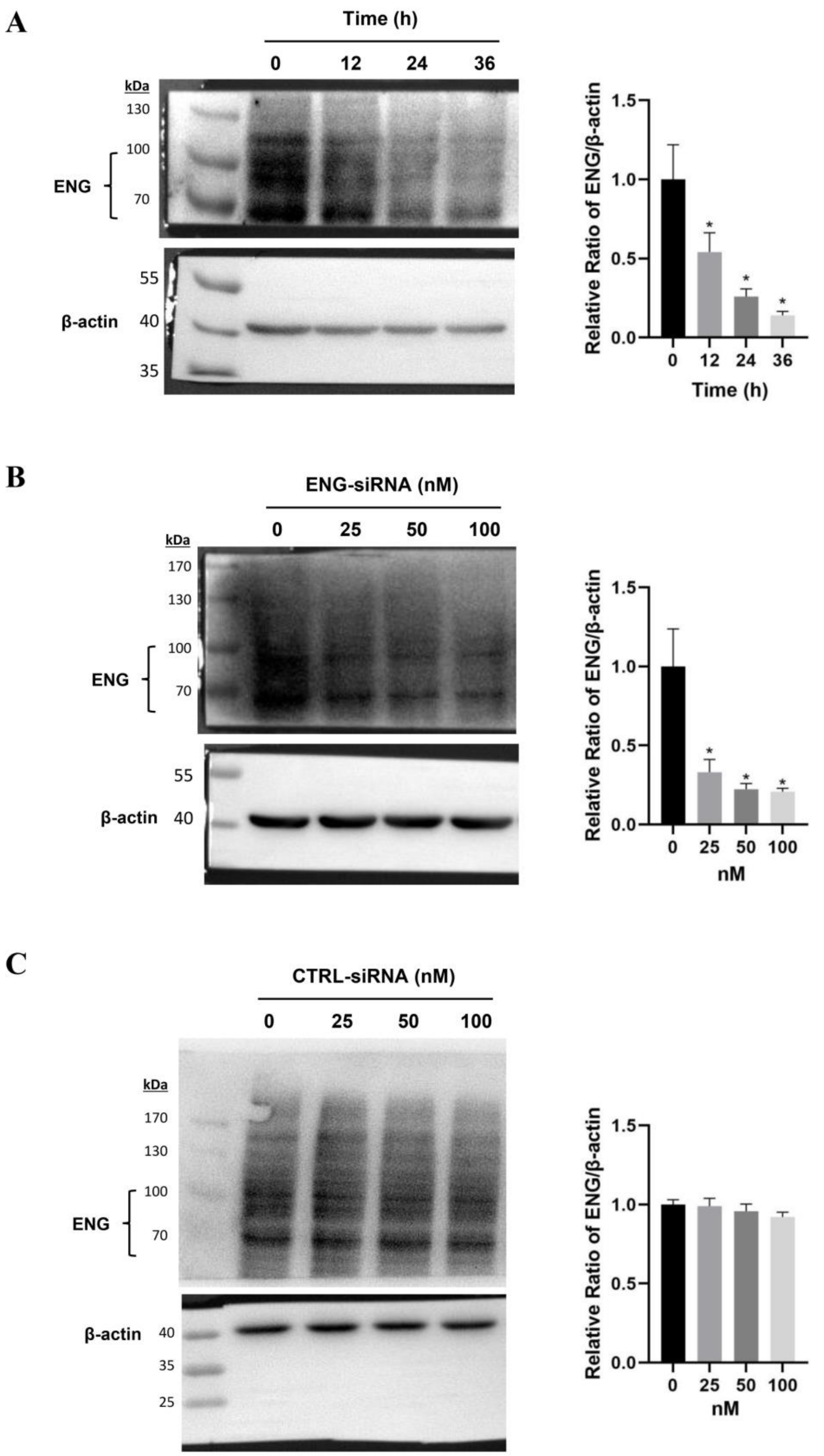

Figure S1. Knockdown efficiency of ENG-small interfering RNA.

Figure 6B

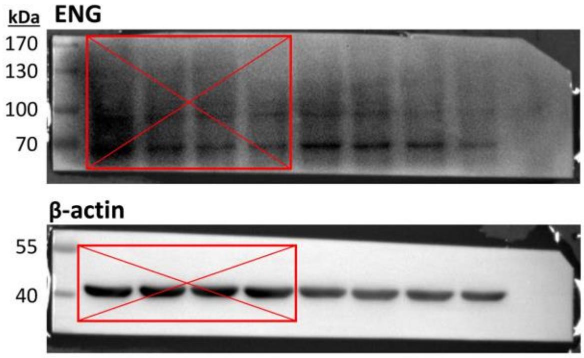

Figure 8A

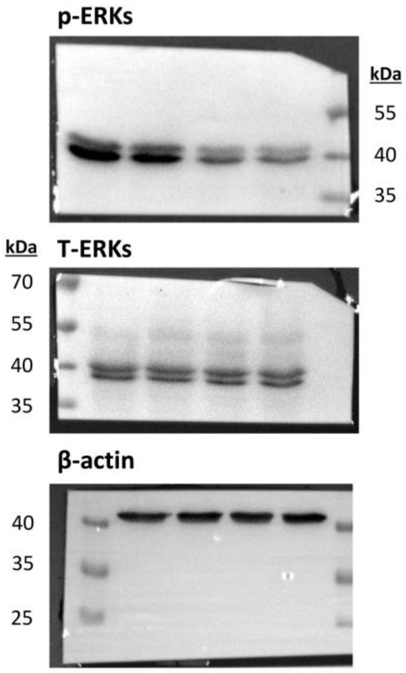

Figure 8B

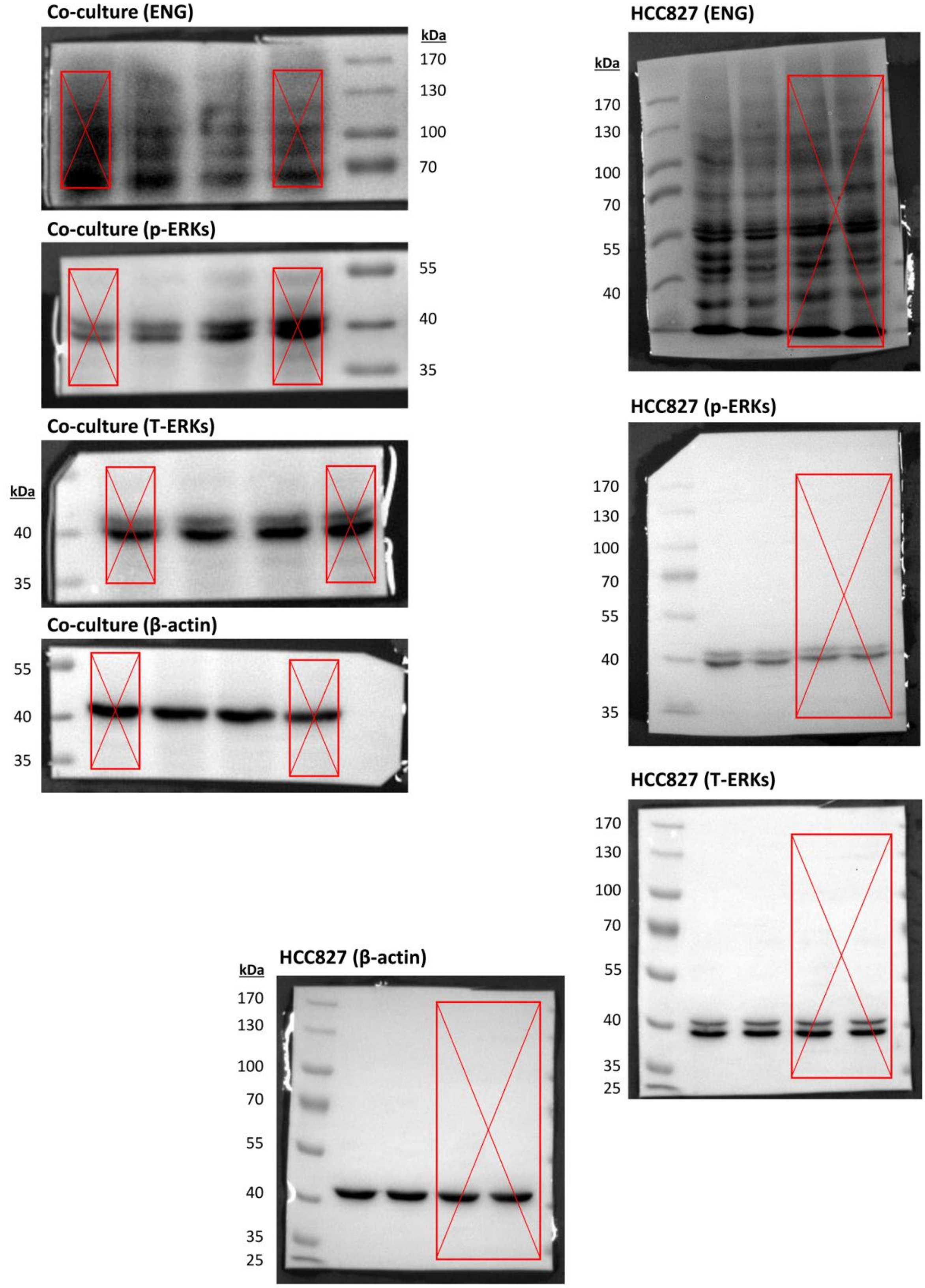
Co-culture (ENG)
kDa
170
130
100
70
Co-culture (p-ERKs)
55
40
35
Co-culture (T-ERKs)
kDa
40
35
Co-culture (β-actin)
55
40
35
HCC827 (ENG)
kDa
170
130
100
70
55
40
HCC827 (p-ERKs)
170
130
100
70
55
40
35
HCC827 (T-ERKs)
170
130
100
70
55
40
35
25
HCC827 (β-actin)
kDa
170
130
100
70
55
40
35
25

Figure 8C

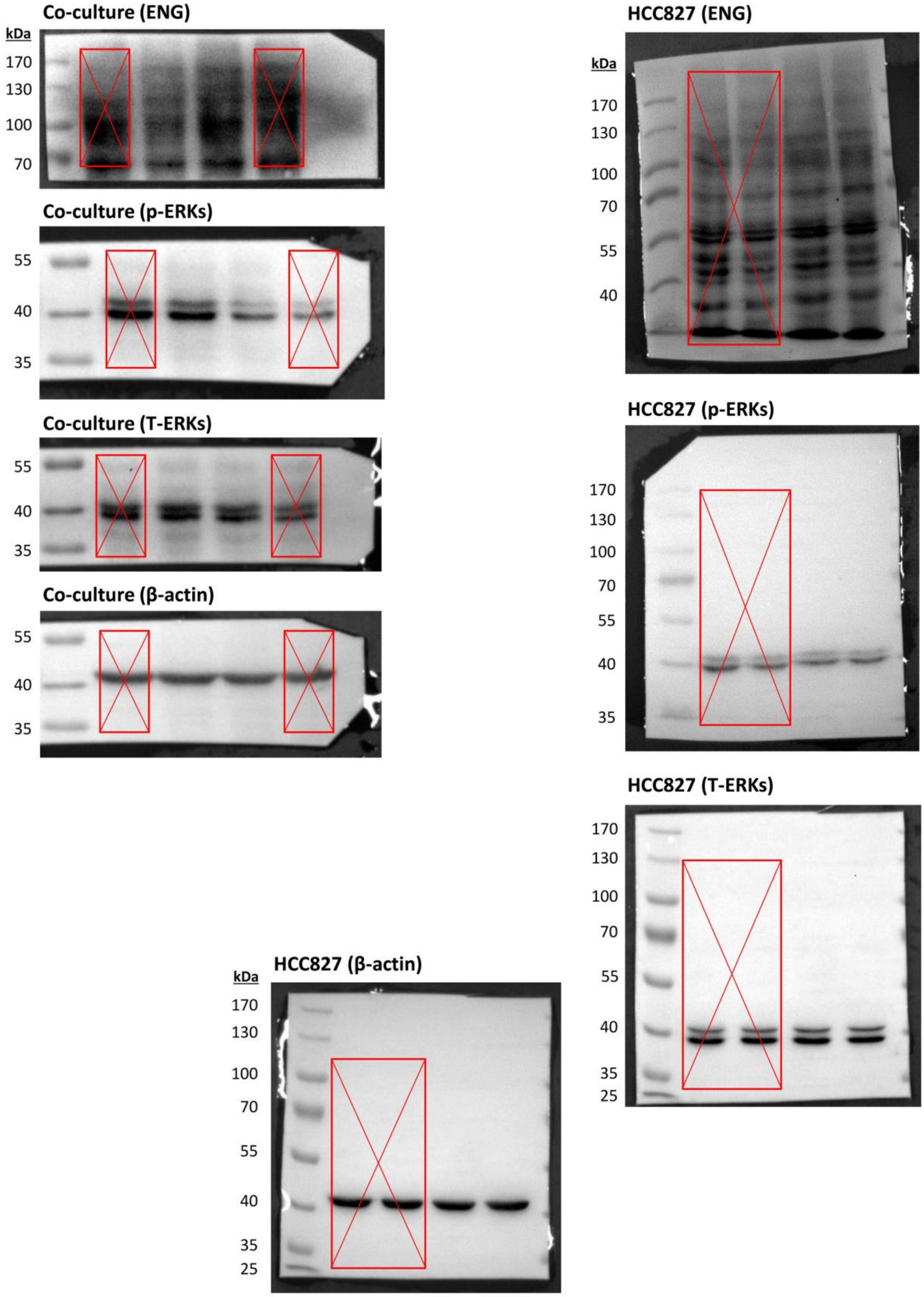

Figure S2. Whole blot showing all the bands with all molecular weight markers on the Western.

© 2020 by the authors. Licensee MDPI, Basel, Switzerland. This article is an open access article distributed under the terms and conditions of the Creative Commons Attribution (CC BY) license (http://creativecommons.org/licenses/by/4.0/).
